# The dopamine D2 agonist quinpirole impairs frontal mismatch responses to sound frequency deviations in freely moving rats

**DOI:** 10.1002/npr2.12199

**Published:** 2021-07-23

**Authors:** Hiroyoshi Inaba, Hisaaki Namba, Satoshi Kida, Hiroyuki Nawa

**Affiliations:** ^1^ Department of Molecular Neurobiology Brain Research Institute Niigata University Niigata Japan; ^2^ Department of Applied Biological Chemistry Graduate School of Agriculture and Life Sciences The University of Tokyo Tokyo Japan; ^3^ Department of Physiological Sciences School of Pharmaceutical Sciences Wakayama Medical University Wakayama Japan

**Keywords:** animal model, dopamine, middle‐latency response, mismatch negativity, schizophrenia

## Abstract

**Aim:**

A reduced mismatch negativity (MMN) response is a promising electrophysiological endophenotype of schizophrenia that reflects neurocognitive impairment. Dopamine dysfunction is associated with symptoms of schizophrenia. However, whether the dopamine system is involved in MMN impairment remains controversial. In this study, we investigated the effects of the dopamine D2‐like receptor agonist quinpirole on mismatch responses to sound frequency changes in an animal model.

**Methods:**

Event‐related potentials were recorded from electrocorticogram electrodes placed on the auditory and frontal cortices of freely moving rats using a frequency oddball paradigm consisting of ascending and equiprobable (ie, many standards) control sequences before and after the subcutaneous administration of quinpirole. To detect mismatch responses, difference waveforms were obtained by subtracting nondeviant control waveforms from deviant waveforms.

**Results:**

Here, we show the significant effects of quinpirole on frontal mismatch responses to sound frequency deviations in rats. Quinpirole delayed the frontal N18 and P30 mismatch responses and reduced the frontal N55 MMN‐like response, which resulted from the reduction in the N55 amplitude to deviant stimuli. Importantly, the magnitude of the N55 amplitude was negatively correlated with the time of the P30 latency in the difference waveforms. In contrast, quinpirole administration did not clearly affect the temporal mismatch responses recorded from the auditory cortex.

**Conclusion:**

These results suggest that the disruption of dopamine D2‐like receptor signaling by quinpirole reduces frontal MMN to sound frequency deviations and that delays in early mismatch responses are involved in this MMN impairment.

## Abstract 2

The subcutaneous administration of quinpirole delayed early mismatch response latencies and reduced a late MMN‐like response amplitude recorded from the frontal cortex but had no effect on those recorded from the auditory cortex. These observations suggest that increased dopamine D2‐like receptor signaling impairs MMN generation to sound frequency changes in the frontal cortex and that the neurochemical mechanisms of MMN vary according to the cortical area. As MMN is associated with cognitive function, these new findings may help develop treatment modalities for cognitive dysfunctions in schizophrenia.

## Graphical abstract

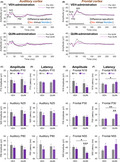

## 1 INTRODUCTION

The pre‐attentive auditory change‐detection system can be tested using mismatch negativity (MMN), a neurophysiological measure.^1^, ^2^ This event‐related potential (ERP) is calculated from electroencephalogram using oddball paradigms consisting of regular patterns of standard sounds and rare deviations (eg, changes of sound frequency). MMN is generated by sources in the auditory and frontal cortices, and these MMN components are associated with the detection of sound changes and the automatic switching of attention, respectively.^3^, ^4^ Numerous clinical studies stating that patients with schizophrenia show reduced MMN amplitudes have made this response a potential neurophysiological biomarker for schizophrenia.^5^, ^6^, ^7^ Cognitive dysfunction is a core feature of schizophrenia and impairs social functioning.^8^, ^9^, ^10^ Currently, no definitive cure exists for the cognitive impairments in schizophrenia. Therefore, uncovering the neural mechanisms underlying these impairments is a major goal of schizophrenia research to develop treatment and prevention strategies. Since MMN deficits are associated with cognitive dysfunction,^11^ elucidating the neural mechanisms of MMN leads to understanding of the cognitive impairments.

Dopamine dysregulation is associated with schizophrenia.^12^ For example, antipsychotics alleviate symptoms of schizophrenia primarily through their inhibitory effects on dopamine D2 receptors.^13^, ^14^ Some antipsychotics ameliorate MMN deficits.^15^, ^16^ In contrast, ketamine administration reduces MMN amplitudes.^17^ This reduction may be due in part to the hyperactivation of the dopamine system because ketamine acts as an agonist of dopamine D2 receptors in addition to an antagonist of N‐methyl‐D‐aspartate (NMDA) receptors.^18^ Therefore, dopamine may regulate MMN; however, this hypothesis is still controversial. Until now, few studies have directly investigated this issue. The administration of the dopamine D2 receptor antagonist haloperidol has been shown to increase MMN and accelerate magnetic MMN.^19^, ^20^ In contrast, subsequent studies on dopamine agonists or antagonists have shown no effect of these drugs on MMN.^21^, ^22^, ^23^

Small rodents, such as mice and rats show MMN‐like responses that closely resemble that of humans.^24^, ^25^, ^26^, ^27^ These animals, which are available for interventional studies, are useful in revealing the molecular and neural bases of MMN.^28^, ^29^ MMN reflects the function of NMDA receptors.^30^ This is supported by the fact that NMDA receptor agonists and antagonists modulate MMN‐like responses in animals.^31^, ^32^, ^33^, ^34^ However, MMN research on the role of the dopaminergic system using animal models has not progressed at all. Thus, this study investigates whether increased dopamine D2‐like receptor signaling impairs mismatch responses generated in the auditory and frontal cortices. To achieve this goal, we examined the effects of the dopamine D2‐like receptor agonist quinpirole on mismatch responses to sound frequency deviations in freely moving rats.

## 2 METHODS

### 2.1 Animals

Male Sprague‐Dawley rats (n = 10; Japan SLC) were housed with 2‐3 per cage in the Niigata University Animal Facility under a reversed 12‐h light/dark cycle (8:00 am OFF and 20:00 pm ON) at constant temperature and humidity. Solid food and water were available ad libitum. All animal experiments were approved by the Animal Care and Use Committee of Niigata University and performed in accordance with the Guide for the Care and Use of Laboratory Animals of the Japan Neuroscience Society.

### 2.2 Surgery and electrode placement

Surgery and electrode placement were performed as described previously.^25^, ^26^, ^27^ Rats (11‐12 weeks old) were anesthetized by intraperitoneal injection of a combination anesthetic containing 0.375‐mg/kg medetomidine (Kyoritsu Seiyaku), 2‐mg/kg midazolam (Sandoz), and 2.5‐mg/kg butorphanol (Meiji Seika Pharma), and positioned in a stereotaxic frame (SR‐5R‐HT; Narishige). Four miniature stainless steel bolts (1.0‐mm diameter) were used as electrodes for recording electrocorticography (ECoG). The bolts were screwed to the skull to contact the dura mater at the following points: the right primary auditory cortex (4.5‐mm posterior to bregma; 8.0‐mm lateral to the midline; 4.2‐mm below the top‐plane of the skull), the right frontal cortex (2.0‐mm posterior to bregma; 2.0‐mm lateral to the midline), the frontal sinus (as a reference, 10.0‐mm anterior to bregma, 1.0‐mm lateral to the midline), and the cerebellum (as a ground, 11.0‐mm posterior to bregma, 1.0‐mm lateral to the midline).^35^ Special attention was paid to minimize the injury of the temporalis muscle; a hemispherical incision was made along the muscle fibers with a radio knife (PROG‐DS3M; J. Morita Tokyo Mfg.), and bleeding was arrested by oxidized cellulose (Surgicel^R^; Ethicon). All lead wires from the electrodes were soldered onto a miniature connector. The connector was anchored to the cranium using dental cement (Quick Resin; Shofu). Rats received cefmetazole (100 mg/kg, ip; Daiichi Sankyo) after surgery. Solid foods were replaced with softened food pellets (CMF sprout; Oriental Yeast) to promote normal weight gain. Recording began at least 14 days after recovery from surgery.

### 2.3 ECoG recording

ECoG recording was performed as described previously.^26^, ^27^ Rats were handled for five consecutive days and acclimatized to a recording chamber prior to the commencement of ECoG recording. ECoG recording occurred in a dimly lit room (0.1 lx) during the dark cycle, which is the active phase in rats.^25^, ^26^, ^27^, ^36^ Each awake rat was placed in the transparent electrically shielded plastic box (dimensions 18 × 36 × 30 cm) during acclimation (15 min) and subsequent stimulation sessions. ECoG signals from the electrodes were fed by wire into an amplifier (1000‐fold; DAM80; World Precision Instruments) and band‐pass filtered at 0.1‐1000 Hz. The amplified signals were digitized and recorded at a 4‐kHz sampling rate (Digidata 1200B and Axoscope v1.1; Axon Instruments).

### 2.4 Stimulus presentation and drug administration

Stimulus presentation was basically performed as described previously.^25^ Auditory stimuli (100‐ms pure tones including 3‐ms rise and fall times) were generated by LabVIEW 2016 (National Instruments) and delivered thorough a full‐range speaker mounted above the recording chamber. The maximum sound pressure level was set to 85 dB at the center of the chamber floor using a sound level meter (33‐2055; RadioShack; set to C‐weighting, fast and max). These tones were presented in a frequency oddball paradigm consisting of ascending and equiprobable (ie, many standards) control conditions.^36^, ^37^, ^38^ In the ascending condition, standard stimuli (3 kHz, 90% probability) and deviant stimuli (6 kHz, 10% probability) were presented a total of 2000 times in pseudo‐random order with a stimulus onset asynchrony of 300 ms In the equiprobable condition, 10 different frequency tones (1, 1.5, 2, 3, 4, 5, 6, 7, 7.5, and 8 kHz, 10% probability each) were presented in the same way as the oddball condition.^25^ In this condition, the 6‐kHz tones were defined as nondeviant stimuli.

The oddball paradigm was comprised of four presentation blocks. In the first block, the ascending or equiprobable condition was used. In the second block, after a 3‐min cessation, another condition that was not used in the first block was applied. Immediately after the second block, rats were injected subcutaneously with vehicle (0.9% NaCl) or quinpirole (0.3 mg/kg; Q102, Sigma‐Aldrich) solution. In the third and fourth blocks 10 min after the administration, stimulus presentations were performed in the same order as before the administration. Three days later, the same rats were subjected to the oddball paradigm again in the same order of the stimulus conditions with another solution that was not administered in the last time. The order of stimulus conditions and solution administration were counterbalanced across animals.

### 2.5 Data analysis

Data analyses were performed offline using MATLAB R2017b (Mathworks). Acquired data were imported into the MATLAB toolbox EEGLAB v13.6.5b (SCCN)^39^ via Spike2 v8.14 (CED) and then downsampled to 1 kHz. The data were offline‐filtered at 0.5‐100 Hz. Epochs were extracted from 100‐ms pre‐ to 300‐ms poststimulus onset for each deviant tone. Only epochs to deviants preceded by at least two standards were extracted.^40^ In the equiprobable condition, all nondeviant epochs were extracted. Epochs containing amplitudes exceeding ± 500 μV at either electrode were rejected as artifacts.^26^, ^27^ The mean (±SEM) number of epochs used for the calculations as follows: pre‐administration (vehicle‐deviant, 185.90 ± 2.34; vehicle‐nondeviant, 183.00 ± 5.28; quinpirole‐deviant, 179.90 ± 4.05; quinpirole‐nondeviant, 182.60 ± 4.24); post‐administration (vehicle‐deviant, 178.60 ± 5.54; vehicle‐nondeviant, 185.60 ± 3.49; quinpirole‐deviant, 185.80 ± 3.04; quinpirole‐nondeviant, 187.80 ± 5.55).

ERP waveforms were calculated separately for deviant and nondeviant stimuli within each animal by averaging the epochs and subtracting the mean value of the 0‐20 ms before stimulus onset.^26^, ^27^, ^41^, ^42^ Difference waveforms were obtained by subtracting the nondeviant ERP from the deviant ERP within each animal. Peak amplitudes and their latencies were measured in the following time windows: 9‐15 ms (auditory P10), 15‐50 ms (auditory N25), 50‐110 ms (auditory P80), 16‐20 ms (frontal N18), 20‐40 ms (frontal P30), and 40‐80 ms (frontal N55) after stimulus onset.

### 2.6 Statistical analysis

Statistical analyses were performed using R v2.8.1 (The R Foundation) and the modified R‐commander v1.4‐8 (a graphical user interface; Department of Physical Therapy, Hirosaki University School of Health Sciences, https://personal.hs.hirosaki‐u.ac.jp/pteiki/research/stat/R/).^43^ Data of peak amplitudes and their latencies were analyzed by two‐factor repeated measures analysis of variance (two‐way ANOVA) with Greenhouse–Geisser correction to identify main effects (drug, time, and stimulus type) and interaction (drug × time and time × stimulus type). If the *P*‐value for the interaction was <.05, the post hoc Shaffer's modified sequentially rejective Bonferroni multiple comparison test was performed. Correlations between ERP components were assessed by Spearman's rank correlation coefficient. A *P*‐value <.05 was considered to be statistically significant.

## 3 RESULTS

Quinpirole could alter auditory‐evoked ERPs, regardless of the deviations, by affecting carrier frequency‐specific responses and the phenomenon of reduction of neuronal responses to repeated stimuli called stimulus‐specific adaptation.^44^ Therefore, to detect “true” MMN‐like response amplitudes to sound frequency deviations, we used the equiprobable control sequence and compared difference waveforms of deviant ERPs minus nondeviant ERPs before and after administration.^25^, ^37^, ^38^ In addition, the vehicle control condition was set considering the possibility that ERPs may change with administration action and time.

### 3.1 MMN‐like difference ERPs recorded from the auditory and frontal cortices

**FIGURE 1:**
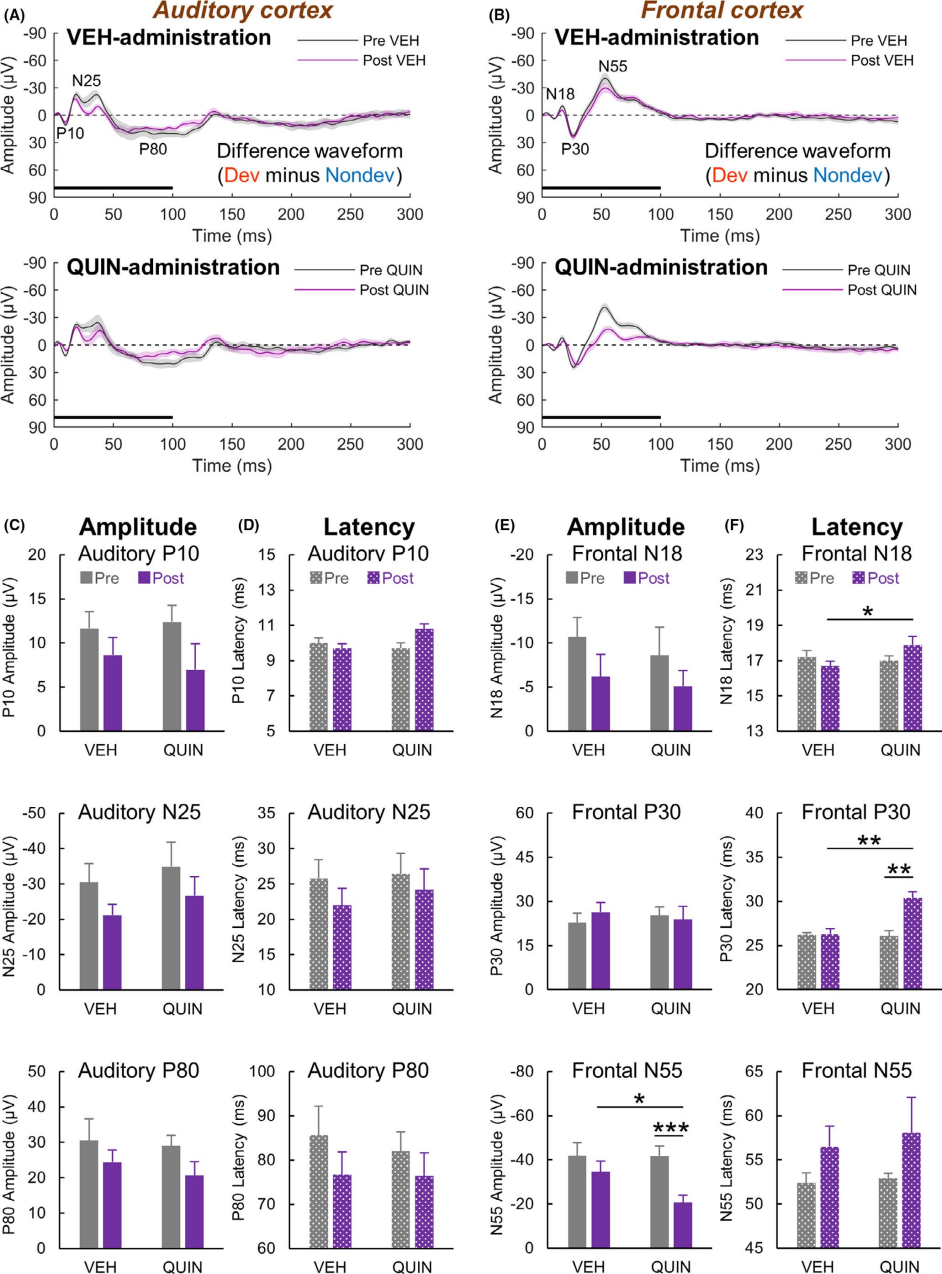
Effects of quinpirole on mismatch responses recorded from the auditory and frontal cortices of freely moving rats. (A, B) Grand averaged difference waveforms obtained by subtracting nondeviant (Nondev) waveforms from deviant (Dev) waveforms before and after vehicle (VEH) and quinpirole (QUIN) administration. Black lines denote stimulus presentation duration. (C, D) Auditory P10, N25, and P80 component peak amplitudes and their latencies from difference waveforms. (E, F) Frontal N18, P30, and N55 component peak amplitudes and their latencies from difference waveforms. Shaded areas around waveforms and error bars denote SEM, n = 10; *P < .05, **P < .01, ***P < .001 (Shaffer's post hoc test)

Figure 1A,B illustrate the averaged difference waveforms recorded from the auditory and frontal cortices before and after vehicle or 0.3‐mg/kg quinpirole administration.

#### 3.1.1 Peak amplitude recorded from the auditory cortex

Two‐way ANOVA for P10, N25, and P80 component amplitudes showed no significant drug (vehicle vs. quinpirole) × time (before vs. after drug administration) interaction (P10: *Drug*, F(1, 9) = 0.06, *P* = .81; *Time*, F(1, 9) = 3.56, *P* = .092; *Interaction*, F(1, 9) = 0.32, *P* = .59; N25: *Drug*, F(1, 9) = 1.45, *P* = .26; *Time*, F(1, 9) = 10.32, *P* = .011; *Interaction*, F(1, 9) = 0.03, *P* = .87; P80: *Drug*, F(1, 9) = 0.31, *P* = .59; *Time*, F(1, 9) = 15.52, *P* = .0034; *Interaction*, F(1, 9) = 0.09, *P* = .77; Figure 1C).

#### 3.1.2 Peak latency recorded from the auditory cortex

Two‐way ANOVA for P10, N25, and P80 component latencies showed no significant drug × time interaction (P10: *Drug*, F(1, 9) = 2.09, *P* = .18; *Time*, F(1, 9) = 2.94, *P* = .12; *Interaction*, F(1, 9) = 4.59, *P* = .061; N25: *Drug*, F(1, 9) = 0.25, *P* = .63; *Time*, F(1, 9) = 1.55, *P* = .24; *Interaction*, F(1, 9) = 0.13, *P* = .73; P80: *Drug*, F(1, 9) = 0.10, *P* = .76; *Time*, F(1, 9) = 2.84, *P* = .13; *Interaction*, F(1, 9) = 0.20, *P* = .66; Figure 1D).

#### 3.1.3 Peak amplitude recorded from the frontal cortex

Two‐way ANOVA for N18 and P30 component amplitudes showed no significant drug × time interaction (N18: *Drug*, F(1, 9) = 2.34, *P* = .16; *Time*, F(1, 9) = 4.60, *P* = .061; *Interaction*, F(1, 9) = 0.03, *P* = .86; P30: *Drug*, F(1, 9) < 0.01, *P* = 1.0; *Time*, F(1, 9) = 0.14, *P* = .72; *Interaction*, F(1, 9) = 1.18, *P* = .31). In contrast, two‐way ANOVA for N55 component amplitudes showed a significant drug ×time interaction (*Drug*, F(1, 9) = 4.69, *P* = .059; *Time*, F(1, 9) = 14.22, *P* = .0044; *Interaction*, F(1, 9) = 5.19, *P* = .049). Shaffer's post hoc test indicated that the N55 amplitude in the post‐quinpirole group was significantly smaller than that in both the post‐vehicle (*P* = .014) and pre‐quinpirole groups (*P* < .001) (Figure 1E).

#### 3.1.4 Peak latency recorded from the frontal cortex

Two‐way ANOVA for N18 and P30 component latencies showed a significant drug × time interaction (N18: *Drug*, F(1, 9) = 2.14, *P* = .18; *Time*, F(1, 9) = 0.22, *P* = .65; *Interaction*, F(1, 9) = 10.76, *P* = .0095; P30: *Drug*, F(1, 9) = 18.00, *P* = .0022; *Time*, F(1, 9) = 9.35, *P* = .014; *Interaction*, F(1, 9) = 12.44, *P* = .0064). Shaffer's post hoc test indicated that the N18 latency in the post‐quinpirole group was significantly longer than that in the post‐vehicle group (*P* = .013), and the P30 latency in the post‐quinpirole group was significantly longer than that in both the post‐vehicle (*P* = .0010) and pre‐quinpirole groups (*P* = .0041). In contrast, two‐way ANOVA for N55 component latencies showed no significant drug × time interaction (*Drug*, F(1, 9) = 0.17, *P* = .69; *Time*, F(1, 9) = 2.77, *P* = .13; *Interaction*, F(1, 9) = 0.08, *P* = .78; Figure 1F).

These results suggested that the quinpirole administration delayed the frontal N18 and P30 mismatch components and reduced the frontal N55 mismatch component. To confirm whether these results in difference waveforms were attributed to quinpirole‐induced changes in deviant ERPs, we examined the effect of drug administration on deviant and nondeviant ERPs before subtraction.

### 3.2 Deviant and nondeviant ERPs recorded from the frontal cortex

**FIGURE 2:**
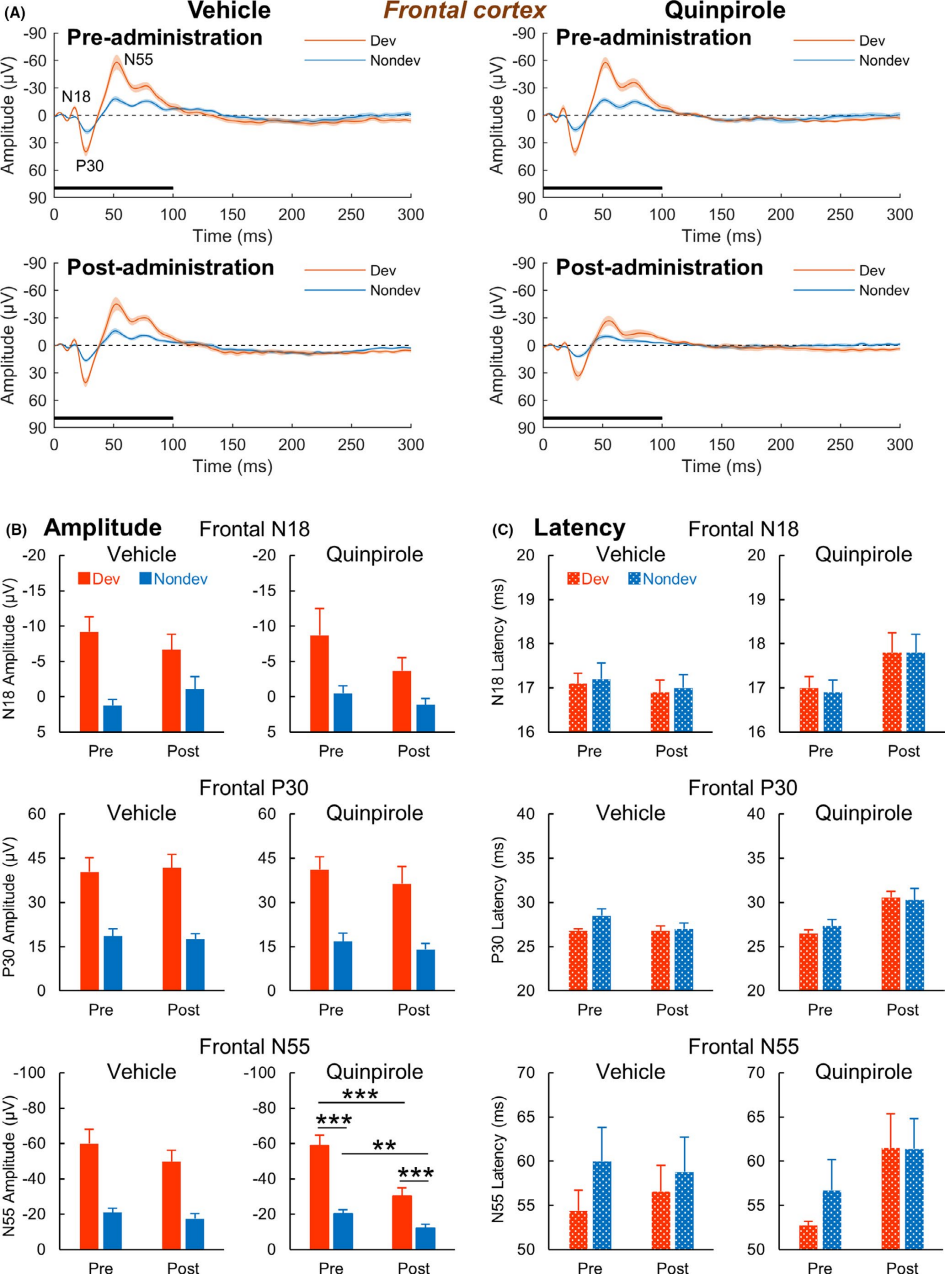
Effects of quinpirole on sound‐evoked potentials recorded from the frontal cortex of freely moving rats. (A) Grand averaged waveforms evoked by deviant (Dev) and nondeviant (Nondev) stimuli before and after vehicle and quinpirole administration. Black lines denote stimulus presentation duration. (B, C) Frontal N18, P30, and N55 component peak amplitudes and their latencies evoked by Dev and Nondev stimuli. Shaded areas around waveforms and error bars denote SEM, n = 10; **P < .01, ***P < .001 (Shaffer's post hoc test)

Figure 2A illustrates the averaged deviant and nondeviant waveforms recorded from the frontal cortex before and after vehicle or 0.3‐mg/kg quinpirole administration (See Figure S1 and Supplemental Results for deviant and nondeviant waveforms recorded from the auditory cortex).

#### 3.2.1 Peak amplitude

Two‐way ANOVA for N18 and P30 component amplitudes showed no significant time (before vs after drug administration) main effect or time × stimulus type (deviant vs nondeviant stimuli) interaction on either drug condition (N18‐vehicle: *Time*, F(1, 9) = 0.00, *P* = .95; *Stimulus type*, F(1, 9) = 27.76, *P* < .001; *Interaction*, F(1, 9) = 1.64, *P* = .23; N18‐quinpirole: *Time*, F(1, 9) = 2.00, *P* = .19; *Stimulus type*, F(1, 9) = 10.15, *P* = .011; *Interaction*, F(1, 9) = 1.33, *P* = .28, P30‐vehicle: *Time*, F(1, 9) = 0.01, *P* = .94; *Stimulus type*, F(1, 9) = 66.34, *P* < .001; *Interaction*, F(1, 9) = 0.47, *P* = .51; P30‐quinpirole: *Time*, F(1, 9) = 0.81, *P* = .39; *Stimulus type*, F(1, 9) = 46.02, *P* < .001; *Interaction*, F(1, 9) = 0.30, *P* = .60). In contrast, two‐way ANOVA for N55 component amplitudes showed a significant time × stimulus type interaction only on the quinpirole condition (vehicle: *Time*, F(1, 9) = 6.26, *P* = .034; *Stimulus type*, F(1, 9) = 56.00, *P* < .001; *Interaction*, F(1, 9) = 1.68, *P* = .23; quinpirole: *Time*, F(1, 9) = 59.35, *P* < .001; *Stimulus type*, F(1, 9) = 70.68, *P* < .001; *Interaction*, F(1, 9) = 33.78, *P* < .001). Shaffer's post hoc test indicated that on the quinpirole condition, significant differences in the N55 amplitude were found between all groups compared (pre‐administration deviant vs post‐administration deviant, *P* < .001; pre‐administration nondeviant vs post‐administration nondeviant, *P* = .0042; pre‐administration deviant vs pre‐administration nondeviant, *P* < .001; post‐administration deviant vs post‐administration nondeviant, *P* < .001; Figure 2B).

#### 3.2.2 Peak latency

Two‐way ANOVA for N18 and P30 component latencies showed a significant time main effect, but not a time × stimulus type interaction, only on the quinpirole condition (N18‐vehicle: *Time*, F(1, 9) = 1.00, *P* = .34; *Stimulus type*, F(1, 9) = 0.23, *P* = .64; *Interaction*, F(1, 9) = 0.00, *P* = 1.0; N18‐quinpirole: *Time*, F(1, 9) = 8.10, *P* = .019; *Stimulus type*, F(1, 9) = 0.04, *P* = .85; *Interaction*, F(1, 9) = 0.04, *P* = .85; P30‐vehicle: *Time*, F(1, 9) = 6.64, *P* = .030; *Stimulus type*, F(1, 9) = 1.65, *P* = .23; *Interaction*, F(1, 9) = 1.71, *P* = .22; P30‐quinpirole: *Time*, F(1, 9) = 40.19, *P* < .001; *Stimulus type*, F(1, 9) = 0.12, *P* = .73; *Interaction*, F(1, 9) = 0.84, *P* = .38). In contrast, two‐way ANOVA for N55 component latencies showed no significant time main effect or time × stimulus type interaction on either drug condition (vehicle: *Time*, F(1, 9) = 0.06, *P* = .82; *Stimulus type*, F(1, 9) = 2.62, *P* = .14; *Interaction*, F(1, 9) = 0.61, *P* = .46; quinpirole: *Time*, F(1, 9) = 3.89, *P* = .080; *Stimulus type*, F(1, 9) = 0.56, *P* = .47; *Interaction*, F(1, 9) = 0.55, *P* = .48; Figure 2C).

These results suggested that the reduction in the frontal N55 mismatch component observed in the difference waveform of the post‐quinpirole group was attributed to the reduction in the deviant ERP, while the delay in the frontal N18 and P30 mismatch components was attributed to the delay in both the deviant and nondeviant ERPs.

### 3.3 Correlation between quinpirole‐affected early and late frontal components

**FIGURE 3:**
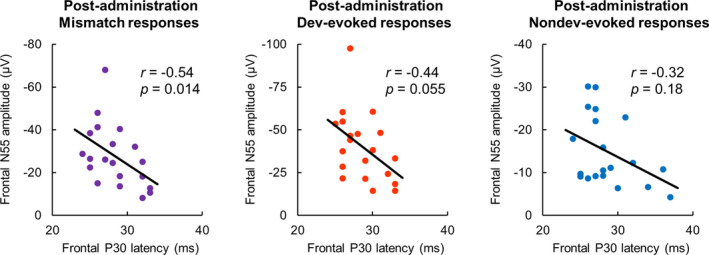
Correlation between amplitude of the frontal mismatch negativity‐like response and latency of the early frontal mismatch response. Frontal N55 amplitudes and frontal P30 latencies used were obtained from difference waveforms (Mismatch), deviant‐evoked waveforms (Dev), and nondeviant‐evoked waveforms (Nondev) after vehicle and quinpirole administration. Correlations were tested with Spearman's correlation coefficient, n = 20 (vehicle, n = 10; quinpirole, n = 10)

We investigated the correlation between quinpirole‐affected ERP components. Spearman's correlation test showed a significant negative correlation between the time of P30 component latency and magnitude of N55 component amplitude, which were obtained from difference waveforms recorded from the frontal electrodes of post‐administered rats (Figure 3; difference, *r* = −.54, *P* = .014; deviant, *r* = −.44, *P* = .055; nondeviant, *r* = −.32, *P* = .18). However, no significant correlation was observed between the N18 latency and N55 amplitude in any of the waveforms (difference, *r* = .20, *P* = .40; deviant, *r* = .27, *P* = .25; nondeviant, *r* = −.31, *P* = .19; data not shown). These results suggested that the latency of the P30 mismatch component was related to the amplitude of the N55 mismatch component.

## 4 DISCUSSION

### 4.1 Principal findings

This study evaluated the effects of the selective dopamine D2‐like receptor agonist quinpirole on MMN‐like responses to sound frequency deviations in freely moving rats. The peak amplitude of deviant ERPs was significantly larger than that of nondeviant ERPs for the frontal components N18, P30, and N55 (Figure 2B). The peak latency of these frontal mismatch components was delayed following quinpirole administration regardless of the stimulus type (Figure 2C). In addition, we observed that the N55 amplitude interacts between time and stimulus type only under the quinpirole condition (Figure 2B). Reflecting on these results, in the difference waveforms, delays in the N18 and P30 latencies and a decrease in the N55 amplitude caused by quinpirole administration were observed (Figure 1E,F). Furthermore, the magnitude of the N55 amplitude was negatively correlated with the time of the P30 latency in difference waveforms (Figure 3). In auditory components P10, N25, and P80, the peak amplitude of deviant ERPs was significantly larger than that of nondeviant ERPs (Figure S1A,B). Furthermore, the peak latency of deviant ERPs was significantly shorter than that of nondeviant ERPs for auditory components P10 and N25 (Figure S1A,C). Therefore, these positive and negative deflections are considered mismatch/MMN‐like responses to sound frequency deviations. In contrast to the mismatch components recorded from the frontal cortex, changes in amplitude and latency of these components following quinpirole administration were similar to those after vehicle administration (Figure S1). Consistently, quinpirole had no discernible effect on these components in deference waveforms (Figure 1C,D), suggesting that disruption of dopamine D2‐like receptor signaling does not affect mismatch responses observed from the auditory cortex. Taken together, it is suggested that excessive dopamine D2‐like receptor signaling reduces frontal MMN by delaying early frontal activities evoked by frequency deviations of sound.

### 4.2 Comparison with previous studies of the role of dopamine in mismatch responses

Several studies have focused on the role of the dopamine system in human MMN.^45^ Kahkonen and Pekkonen et al have shown that dopamine D2 antagonism following acute administration of haloperidol increases frontal MMN and accelerates magnetic MMN to sound frequency changes, while decreasing the awareness/attention‐related potentials (P3a, processing negativity, and reorienting negativity) in healthy volunteers.^19^, ^20^, ^21^ However, Leung et al have shown that dopamine agonism following acute administration of the dopamine D2 agonist bromocriptine and the dopamine D1/D2 agonist pergolide does not affect MMN and P3a to sound duration changes.^22^ The neural networks involved in MMN generation differ between the case of frequency deviation and that of duration deviation.^46^ These findings and the results of this study suggest that the activation of the dopamine D2 system negatively and positively regulates pre‐attentive and awareness/attention processes caused by frequency deviations rather than duration deviations, respectively. However, this hypothesis should be cautiously considered because the administration of methylphenidate, which enhances dopaminergic transmission, and acute dopamine depletion had no effect on MMN and P3a to either frequency or duration changes.^23^, ^47^, ^48^

### 4.3 Limitations of this study

This study has several limitations. First, we measured MMN‐like responses in rats, unlike previous studies that focused on the role of dopamine in human MMN. However, so far, we and other groups have succeeded in measuring true MMN‐like responses to sound frequency deviations from the auditory and/or frontal cortices of freely moving rats.^25^, ^33^, ^36^, ^37^, ^40^ Furthermore, since the frontal N55 component of the rats was attenuated by NMDAR antagonism, this negative component is considered equal to human frontal MMN.^33^ In humans, true deviation detection is observed not only in MMN but also in middle‐latency responses (MLRs).^49^, ^50^ This finding suggests that the auditory deviation detection system is organized hierarchically.^51^ In a study on rats, the early frontal components N18 and P30 with properties similar to human MLRs were observed, as in the results of this study.^33^ Therefore, the neurochemical mechanisms of human MMN and MLR could be discussed from the results of this study.

Second, we only experimented with systemically administering 0.3‐mg/kg quinpirole. Quinpirole induces a biphasic effect on locomotion and wakefulness depending on the dose.^52^, ^53^ Quinpirole increased wakefulness at doses above 0.25 mg/kg and induced schizophrenia‐related behavioral abnormalities, such as hyperlocomotion and impaired reversal learning at a dose of 0.3 mg/kg in rats,^52^, ^54^, ^55^ implying that the dose used in this study enhanced dopamine D2‐like receptor neurotransmission and disturbed cognitive function. However, further studies using different doses may be needed to firmly insist that the results of this study are derived from increased dopamine signaling.

Third, although an interaction between time and stimulation type was observed in the frontal N55 amplitude under the quinpirole condition, no significant difference in reduction rates was observed between the deviant and nondeviant ERP amplitudes before and after the administration of quinpirole (mean ± SEM; deviant, 48% ± 5%; nondeviant, 40% ± 7%). Thus, hyperdopaminergic transmission may disrupt mismatch responses by not only affecting deviation detection but also impairing the auditory‐evoked frontal activity itself. However, the important points in this study are that in any case, a late frontal MMN‐like response was markedly reduced, and an early frontal MLR‐like potential was correlatively delayed. As mentioned above, most human studies to date have not observed the effects of dopaminergic manipulation on the amplitude and latency of mismatch responses.^22^, ^23^ The findings of this study suggest that the effects of dopaminergic manipulation on mismatch responses need to be reconsidered for each deviation type and brain region.

### 4.4 Relationship of dopaminergic status to MMN deficits in schizophrenia

MMN generators are separated into the temporal (auditory cortex) and frontal (prefrontal cortex) regions.^56^ Experimental studies have demonstrated that these temporal and frontal components have sensory memory comparison and attention allocation functions, respectively.^57^, ^58^, ^59^ Interestingly, several studies have shown that MMN impairment in schizophrenia is primarily due to the inactivation of the frontal generator.^60^, ^61^, ^62^ Furthermore, a reduction in MMN recorded from frontal electrodes, but not from temporal electrodes, has been observed in subjects of 22q11.2 deletion syndrome with *catechol‐O‐methyltransferase* ^108/158^ *Met* allele (ie, with low dopamine clearance capacity) at high risk for schizophrenia.^63^ Similar to the results of the present study, we previously showed reduced potential amplitudes and neural oscillations in the frontal, but not temporal, component of MMN to frequency deviations in a cytokine‐challenged rat model,^25^ which exhibits behavioral and neurophysiological endophenotypes of schizophrenia and hyperdopaminergic activity.^27^, ^64^ On the other hand, in a study of the temporal MMN component in a rat model for schizophrenia induced by subchronic administration of phencyclidine, which can mimic symptoms of schizophrenia and activates the mesolimbic dopamine pathway,^65^ responses to frequency deviations were normal (although reduced responses to duration deviations were observed).^32^ These and our results, in which only the frontal MMN component to frequency deviations was impaired, suggest the possibility that dysfunction of involuntary attention switching in the frontal cortex stemming from dopaminergic abnormalities is one of the causes of MMN deficits in schizophrenia.

## 5 CONCLUSION

The subcutaneous administration of quinpirole delayed early mismatch response latencies and reduced a late MMN‐like response amplitude recorded from the frontal cortex but had no effect on those recorded from the auditory cortex. These observations suggest that increased dopamine D2‐like receptor signaling impairs MMN generation to sound frequency changes in the frontal cortex and that the neurochemical mechanisms of MMN vary according to the cortical area. As MMN is associated with cognitive function, these new findings may help develop treatment modalities for cognitive dysfunctions in schizophrenia.

## CONFLICT OF INTEREST

The authors declare no conflict of interest.

## AUTHOR CONTRIBUTIONS

Conception of the study: HI and HNw. Design of the study: HI. Acquisition of data: HI, HNm, and HNw. Analysis of data: HI. Writing the manuscript: HI. Support for the data analysis and manuscript writing: SK. All the authors reviewed, revised, and approved the manuscript before submission.

## ANIMAL STUDIES

All animal experiments were approved by the Animal Care and Use Committee of Niigata University and performed in accordance with the Guide for the Care and Use of Laboratory Animals of the Japan Neuroscience Society. All efforts were made to minimize animal suffering and to reduce the number of animals used.

## Supporting information

Supplementary Material

## Data Availability

The data that support the findings of this study are available from the corresponding author upon reasonable request.
