# Supplementary material for: The dopamine D2 agonist quinpirole impairs frontal mismatch responses to sound frequency deviations in freely moving rats
Source: Neuropsychopharmacol Rep. 2021 Jul 23;41(3):405–15. doi: 10.1002/npr2.12199 (PMC8411315; doi:10.1002/npr2.12199)
Supplement: Supplementary file 1 — Supplementary Material [file NPR2-41-405-s001.pdf]

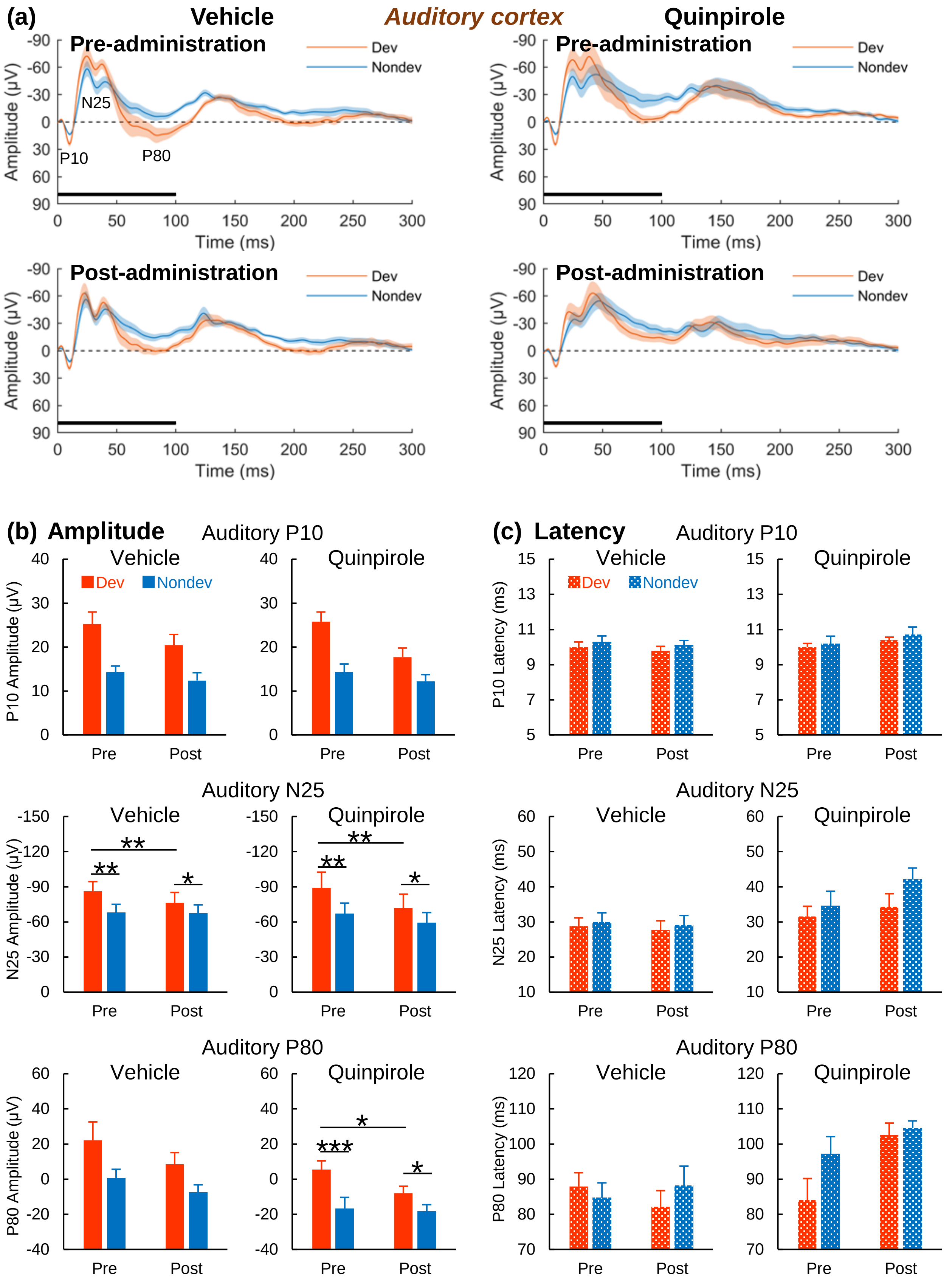

Fig.S1 Effects of quinpirole on sound-evoked potentials recorded from the auditory cortex of freely moving rats. (a) Grand averaged waveforms evoked by deviant (Dev) and nondeviant (Nondev) stimuli before and after vehicle and quinpirole administration. Black lines denote stimulus presentation duration. (b, c) Auditory P10, N25, and P80 component peak amplitudes and their latencies evoked by dev and nondev stimuli. Shaded areas around waveforms and error bars denote *SEM*, $n = 10$; $^{*}P < 0.05$, $^{**}P < 0.01$, $^{***}P < 0.001$ (Shaffer's post-hoc test).

SUPPLEMENTAL RESULTS

Deviant and nondeviant waveforms recorded from the auditory cortex (Fig. S1)

Amplitude

P10-vehicle: *Time*, *F*(1, 9) = 17.39, *P* = 0.0024; *Stimulus type*, *F*(1, 9) = 40.74, *P* < 0.001; *Interaction*, *F*(1, 9) = 1.26, *P* = 0.29; P10-quinpirole: *Time*, *F*(1, 9) = 7.63, *P* = 0.022; *Stimulus type*, *F*(1, 9) = 28.17, *P* < 0.001; *Interaction*, *F*(1, 9) = 3.22, *P* = 0.11; N20-vehicle: *Time*, *F*(1, 9) = 13.08, *P* = 0.0056; *Stimulus type*, *F*(1, 9) = 12.14, *P* = 0.0069; *Interaction*, *F*(1, 9) = 9.96, *P* = 0.012; N20-quinpirole: *Time*, *F*(1, 9) = 11.75, *P* = 0.0075; *Stimulus type*, *F*(1, 9) = 10.18, *P* = 0.011; *Interaction*, *F*(1, 9) = 7.41, *P* = 0.023 (*Post-hoc tests*, pre-administration deviant vs. post-administration deviant, vehicle: *P* = 0.0035, quinpirole: *P* = 0.0039; pre-administration deviant vs. pre-administration nondeviant, vehicle: *P* = 0.0032, quinpirole: *P* = 0.0069; post-administration deviant vs. post-administration nondeviant, vehicle: *P* = 0.041, quinpirole: *P* = 0.032); P80-vehicle: *Time*, *F*(1, 9) = 13.04, *P* = 0.0057; *Stimulus type*, *F*(1, 9) = 13.29, *P* = 0.0054; *Interaction*, *F*(1, 9) = 1.43, *P* = 0.26; P80-quinpirole: *Time*, *F*(1, 9) = 3.31, *P* = 0.10; *Stimulus type*, *F*(1, 9) = 42.12, *P* < 0.001; *Interaction*, *F*(1, 9) = 13.11, *P* = 0.0056 (*Post-hoc tests*, pre-administration deviant vs. post-administration deviant, *P* = 0.026; pre-administration deviant vs. pre-administration nondeviant, *P* < 0.001; post-administration deviant vs. post-administration nondeviant, *P* = 0.012).

Latency

P10-vehicle: *Time*, *F*(1, 9) = 2.25, *P* = 0.17; *Stimulus type*, *F*(1, 9) = 7.36, *P* = 0.024; *Interaction*, *F*(1, 9) = 0.00, *P* = 1.0; P10-quinpirole: *Time*, *F*(1, 9) = 1.43, *P* = 0.26; *Stimulus type*, *F*(1, 9) = 0.79, *P* = 0.40; *Interaction*, *F*(1, 9) = 0.03, *P* = 0.86, N25-vehicle: *Time*, *F*(1, 9) = 5.52, *P* = 0.043; *Stimulus type*, *F*(1, 9) = 9.30, *P* = 0.014; *Interaction*, *F*(1, 9) = 1.33, *P* = 0.28; N25-quinpirole: *Time*, *F*(1, 9) = 5.49, *P* = 0.044; *Stimulus type*, *F*(1, 9) = 11.74, *P* = 0.0076; *Interaction*, *F*(1, 9) = 0.87, *P* = 0.38; P80-vehicle: *Time*, *F*(1, 9) = 0.16, *P* = 0.70; *Stimulus type*, *F*(1, 9) = 0.23, *P* = 0.64; *Interaction*, *F*(1, 9) = 5.56, *P* = 0.043 (*Post-hoc tests*, all *P*s > 0.05); P80-quinpirole: *Time*, *F*(1, 9) = 10.24, *P* = 0.011; *Stimulus type*, *F*(1, 9) = 8.47, *P* = 0.017; *Interaction*, *F*(1, 9) = 4.69, *P* = 0.059.
